# Self-Assembly in ultrahigh molecular weight sphere-forming diblock copolymer thin films under strong confinement

**DOI:** 10.1038/s41598-019-54648-3

**Published:** 2019-12-04

**Authors:** Wei Cao, Senlin Xia, Michael Appold, Nitin Saxena, Lorenz Bießmann, Sebastian Grott, Nian Li, Markus Gallei, Sigrid Bernstorff, Peter Müller-Buschbaum

**Affiliations:** 1 Technische Universität München, Physik-Department, Lehrstuhl für Funktionelle Materialien, James-Franck-Straße 1, 85748 Garching, Germany; 2 Technische Universität Darmstadt, Ernst-Berl-Institute for Technical and Macromolecular Chemistry, Alarich-Weiss-Straße 4, 64287 Darmstadt, Germany; 3 Saarland University, Chair in Polymer Chemistry, Campus C4 2, 66123 Saarbrücken, Germany; 4 Elettra-Sincrotrone Trieste S.C.p.A., Strada Statale 14, km 163.5, in AREA Science Park, 34149 Trieste, Italy; 5 Technische Universität München, Heinz Maier-Leibnitz Zentrum (MLZ), Lichtenbergstr. 1, 85748 Garching, Germany

**Keywords:** Polymers, Self-assembly

## Abstract

Ultrahigh molecular weight (UHMW) diblock copolymers (DBCs) have emerged as a promising template for fabricating large-sized nanostructures. Therefore, it is of high significance to systematically study the influence of film thickness and solvent vapor annealing (SVA) on the structure evolution of UHMW DBC thin films. In this work, spin coating of an asymmetric linear UHMW polystyrene-*block*-poly(methyl methacrylate) (PS-*b*-PMMA) DBC is used to fabricate thin films, which are spherically structured with an inter-domain distance larger than 150 nm. To enhance the polymer chain mobility and facilitate approaching equilibrium nanostructures, SVA is utilized as a post-treatment of the spin coated films. With increasing film thickness, a local hexagonal packing of PMMA half-spheres on the surface can be obtained, and the order is improved at larger thickness, as determined by grazing incidence small angle X-ray scattering (GISAXS). Additionally, the films with locally hexagonal packed half-spherical morphology show a poor order-order-poor order transition upon SVA, indicating the realization of ordered structure using suitable SVA parameters.

## Introduction

Diblock copolymer (DBC) thin films have gained increasing interest in the application of bottom-up nanofabrication, due to their ability to form various periodic structures such as spheres, cylinders, and lamellae resulting from microphase separation^1–7^. So far, most studies have focused on DBCs with low or intermediate molecular weights^8–12^. Due to scaling of the microphase separation structure with respect to the molecular weight of the DBCs, DBCs generally form a characteristic structure size (inter-domain distance) of 10–100 nm^12–16^. However, such nanostructures are limited in their application due to their relatively small inter-domain distances and domain sizes. For example, DBCs with inter-domain distances smaller than 150 nm limit the application of photonic band gap structures in the field of visible light, due to their weak ability for suppressing the reflectivity^15,17^. In addition, small-sized nanodomains do not provide sufficient space for embedding large nanoparticles due to the unfavorable ratio of polymer domain sizes and typical nanoparticle diameters^18^.

Using ultrahigh molecular weight (UHMW) DBCs (number average molecular weight, *M*_n_ > 500 kg/mol) can be an effective approach for achieving a template with large inter-domain distances and domain sizes (i.e., inter-domain distances larger than 150 nm)^14,15^. However, the fabrication of ordered nanostructured films with UHMW DBCs is challenging. UHMW DBCs do not easily approach thermodynamic equilibrium to form ordered structures by microphase separation, due to their very low chain mobility caused by the highly entangled chain conformations^14^. To address this issue, post-treatments, like solvent vapor annealing (SVA) can be employed^19–25^. Through the annealing process, the mobility of the long polymer chains can be effectively enhanced^21–27^. Therefore, the rearrangement of polymer chains into ordered structures can be realized, if sufficient annealing time is applied. The type and size of the periodic nanostructure of UHMW DBCs is significantly influenced by the SVA conditions, such as polymer–solvent interaction parameters (A block-solvent and B block-solvent) as well as the swollen film thickness. Moreover, the final morphology of UHMW DBCs is also determined by the Flory-Huggins segmental interaction parameter (χ), the total number of segments (N) and the composition (volume fraction of block A, *Φ*_A_ and volume fraction of block B, *Φ*_B_)^28–31^.

Recently, Kim *et al*. combined thermal annealing and SVA to achieve ordered large domain sized lamellar nanostructures of polystyrene-*block*-poly(methyl methacrylate) (PS-*b*-PMMA) films^32^. Mapas *et al*. further studied the changes in the morphology (lamellar and cylinder) and domain size with different volume fractions and molecular weights of UHMW poly(solketal methacrylate)-*block*-polystyrene (PSM-*b*-PS)^14^. However, concerning spherical DBC films, most studies only focus on the DBCs with normal (low or intermediate) molecular weights (*M*_n_ < 500 kg/mol)^33–35^. For example, Stein *et al*. studied the morphology evolution of normal molecular weight spherical-domain polystyrene-*b*lock-poly(2 vinylpyridine) (PS-*b*-PVP) as a function of the number of layer (film thickness)^34^. Systematic studies on UHMW sphere-forming DBC films are rarely reported, in particular at highly confined conditions.

Generally, toluene is considered as a good solvent for dissolving PS-*b*-PMMA with sufficient volatility^36,37^. However, toluene is a hydrophobic solvent, which cannot be combined with hydrophilic materials for extending applications. Thus, in the present work, we exploit a hydrophilic solvent instead, namely dimethylformamide (DMF), for dissolving an asymmetric UHMW PS-*b*-PMMA DBC to fabricate ordered large-sized spherical nanostructured thin films. The structural evolution of the fabricated UHMW DBC thin films is systematically investigated as function of increasing film thickness and SVA time. Surface structures of the films are probed via atomic force microscopy (AFM), and the buried structures are detected with grazing incidence small angle X-ray scattering (GISAXS). Results show that an ordered nanostructure with an average half-sphere diameter of around 82 nm and an average inter-domain distance of around 151 nm can be achieved for the studied UHMW DBC thin films through the control of the film thickness and the SVA time.

## Results and Discussion

### Influence of film thickness

In previous studies, it was reported that increasing film thickness to a thin film monolayer, the half-spherical morphology is hexagonal packing in case of a normal molecular weight BCP film^33–35,38^. Moreover, a random island-like morphology is usually observed in thermally equilibrated films, due to the very high confinement effects between the film thickness (t) and inter-domain distance (L_0_). To investigate if these findings can be extended into the regime of UHMW DBCs, films with different film thicknesses have been prepared by spin coating on pre-cleaned substrates. The film thickness is controlled no more than 0.5 L_0_ for achieving a highly confined system.

The film thickness (c > 3.0 mg/mL) is measured by XRR (Fig. S1a). Smaller concentrations (c ≤ 3.0 mg/mL) lead to discontinuous and rough spin coated films (dewetting), which cannot be probed by XRR^39^. Therefore, surface profilometry is utilized as complementary measurement method for these films. Figure S1b shows the thickness of PS-*b*-PMMA thin films at different concentrations. The film thickness increases from 7 ± 2 nm nearly-linear to 91 ± 1.6 nm with increasing PS-*b*-PMMA concentration. Thus, also for UHMW PS-*b*-PMMA DBC the predictions from the spin coating equation are fullfilled^40^.

**Figure 1:**
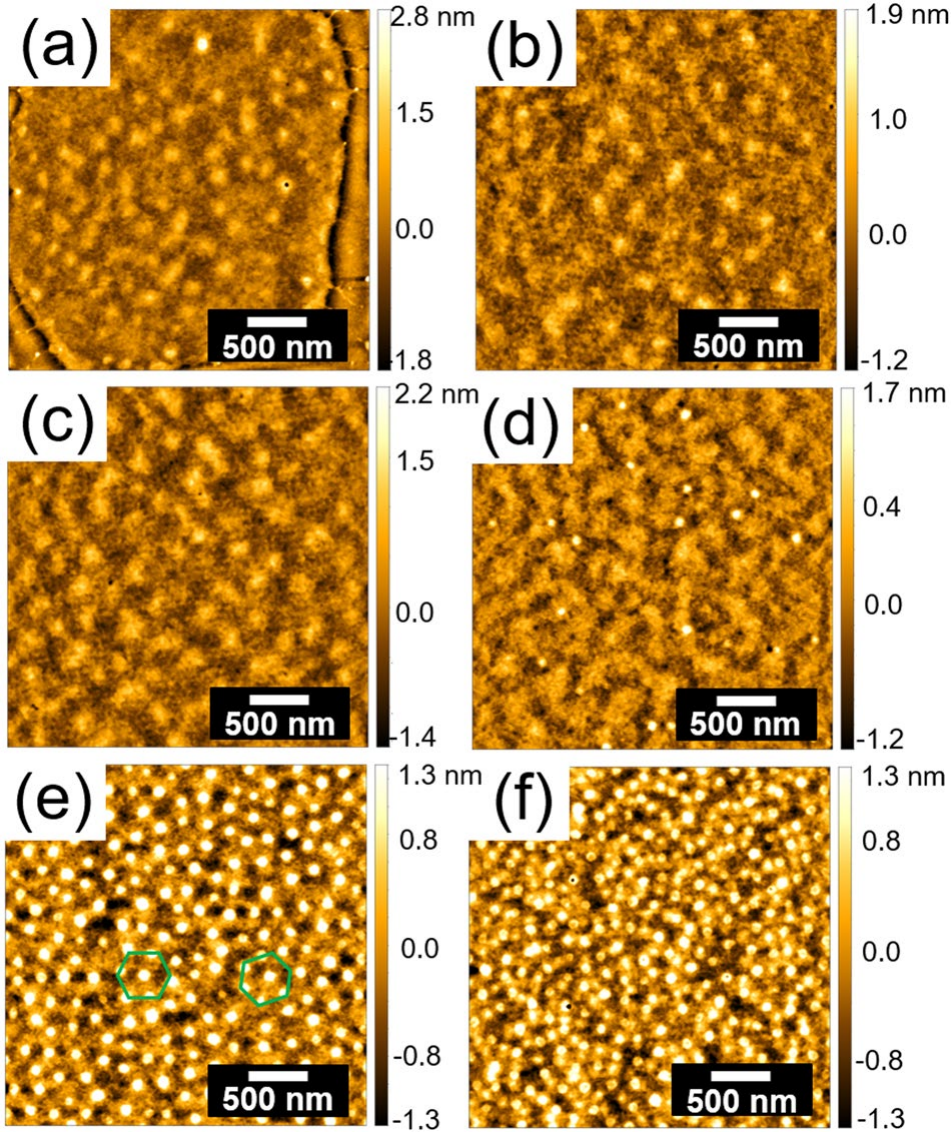
AFM images of PS-*b*-PMMA thin films with thickness of (**a**) 7 nm, (**b**) 12 nm, (**c**) 19 nm, (**d**) 35 nm, (**e**) 56 nm, and (**f**) 91 nm after SVA in THF for 18 h. The height bar is adapted individually as indicated. The green hexagons indicate that the positions of these half-spheres are not well located at the apex of the hexagon.

As seen in Fig. 1, the AFM images show the evolution of the film surface morphology with film thicknesses after SVA (THF, 18.0 h), in which bright spherical domains and a dark matrix are the PMMA and PS block segments, respectively. Relatively ordered nearly half PMMA spheres with a diameter (D) of 87 ± 11 nm (Fig. S3b) and an inter-domain distance of 192 ± 27 nm (Fig. S3e) are observed when the film thickness is 56 nm (Fig. 1e). The half-spherical morphology follows locally a hexagonal packing (“hand”-drawn green hexagons in Figs. 1e and S4e) but is irregular on large scale (ring-like 2D fast Fourier transform pattern in Fig. S4e). Since the film thickness (56 nm) is less than D (diameter of PMMA half-sphere, 82 nm), and higher than 0.5 D (41 nm), the arrangement of a full sphere is not favorable. Moreover, the morphology with half-spheres can maximize the conformational entropy of the polymer chains in the lateral direction^30,41,42^.

For thinner films (7 nm, 12 nm, and 19 nm), a disordered elongated structure is found. No phase separation is found in the corresponding AFM phase images (Fig. S4a–c). This is related to the confinement effects between the film thickness and inter-domain distance, because the films are too thin to form a layer with spherical nanostructure^43–47^. Moreover, the effect of dewetting cannot be ignored, especially for the thinnest film (7 nm)^48^. A film edge (dark line) is seen in local areas in Fig. 1a, indicating insufficient surface coverage and island formation^49^. As the film thickness increases to 35 nm, the elongated structures still exists while some half-spheres appear as well. Partial PMMA half-spheres randomly appear in the film, due to a film thickness which is not sufficient to support the formation of one layer of PMMA half-spheres. Moreover, the thickness’s value is close to the value of the PMMA radius (around 44 nm at a film thickness of 56 nm), which increases the possibility of forming PMMA half-spheres. Figure S3a shows that the obtained PMMA half-spheres in Fig. 1d have a radius of around 39 nm, which is larger than the value of film thickness (35 nm). This indicates that the half-spheres might be compressed in the vertical direction. In contrast, in the thicker film (91 nm, Fig. 1f), the distance between neighboring PMMA half-spheres seems to be reduced as compared with the thickness of 56 nm. However, from the corresponding AFM phase image (Fig. S4f), the distance (Fig. S3f, 191 ± 24 nm) does not change and the spherical size (Fig. S3c, 83 ± 9 nm) is only slightly decreased. Such difference between the height image and phase image might be attributed to the surface defects from PS chains. In thicker film the polymer has sufficient space in the vertical direction to adopt an unperturbed chain conformation. In addition, “hand”-drawn green hexagons and ring-like 2D fast Fourier transform (FFT) pattern in Fig. S4f confirms the only locally regular hexagonal packing of these PMMA half-spheres.

**Figure 2:**
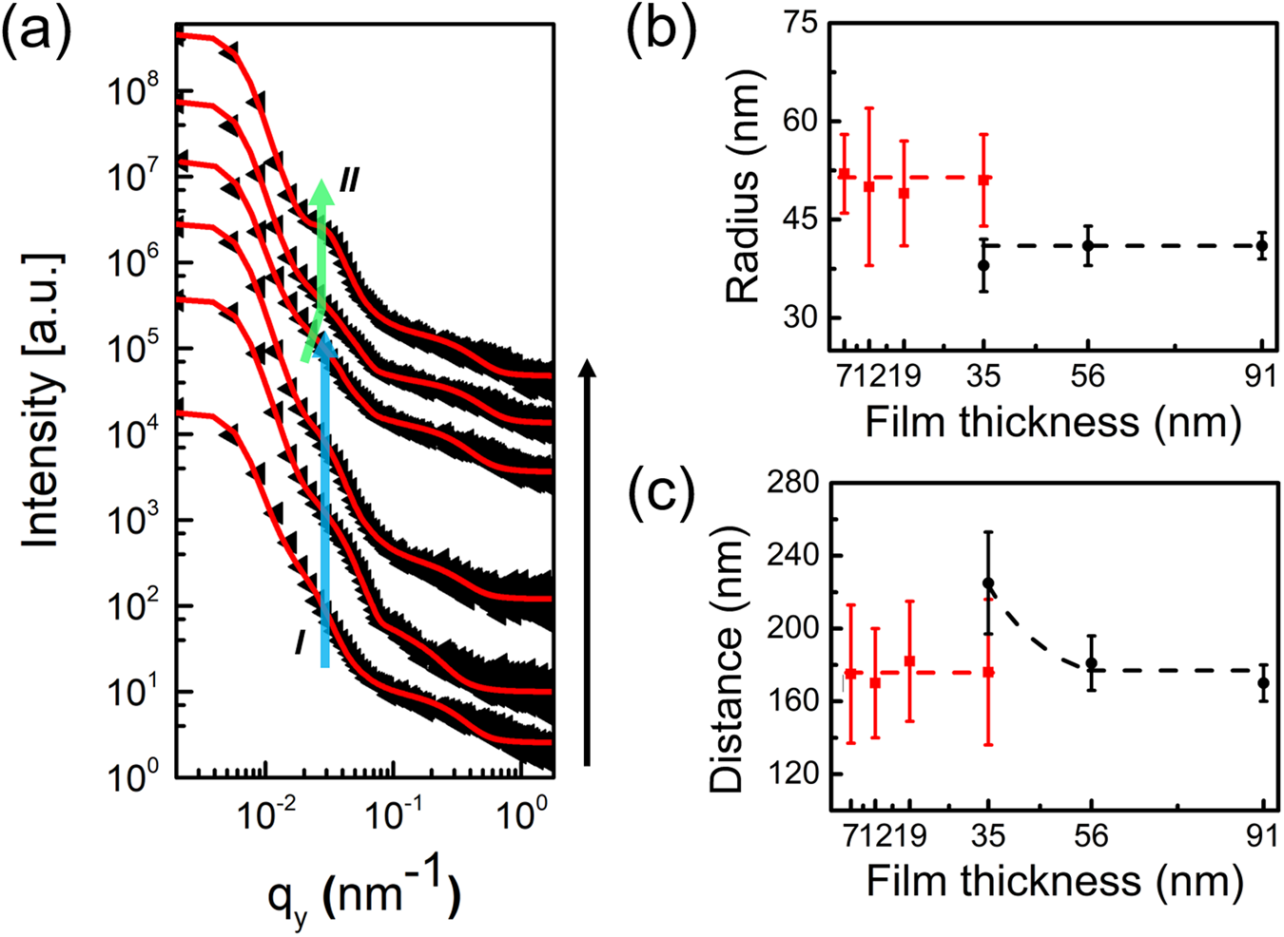
Horizontal line cuts (q_y_) from the 2D GISAXS data of the PS-*b*-PMMA thin films with different thicknesses after SVA in THF for 18 h. (**a**) Data (black dots) and fits (solid red lines) are shifted vertically along the y axis for clarity of the presentation (7 nm, 12 nm, 19 nm, 35 nm, 56 nm, and 91 nm from bottom to top). The blue and green arrows serve as a guide to the eye for the main contributions I and II. (**b**) Radius and (**c**) distance of PMMA elongated structure (red) and PMMA half-spheres (black) determined from the fits of the GISAXS data plotted as a function of film thickness. The dashed lines in (**b**,**c**) serve as a guide to the eye.

In order to study the inner morphology of the films with high statistical relevance, all films are investigated with GISAXS after SVA in THF for 18 h^50^. The large footprint of the X-ray beam, which has its origin in the shallow grazing incident angle, allows for probing a large film volume and therefore yields lateral structure information of the films with high statistical relevance. As shown in Fig. S5, horizontal line cuts of the 2D GISAXS data (Fig. S6) are done at the Yoneda peak position of PS-*b*-PMMA. They reveal lateral structure sizes, as depicted in Fig. 2a. For quantitative analysis, the horizontal line cuts are fitted in the framework of the distorted-wave Born approximation (DWBA) using the effective interface approximation (EIA). In the model a half spherical geometry of the scattering objects is assumed to match the AFM observations, which is based on the ratio of sphere diameter to film thickness^50^. In case of the film thickness of 35 nm, compared to the thinner films (7 nm, 12 nm, 19 nm), an additional half spherically shaped scattering object is needed to fit the data and to account for the presence of half-spheres as seen also in the AFM data. The form factors refer to the radius of the PMMA half-spheres, the structure factors describe the center-to-center distances of neighboring PMMA half-spheres (inter-domain distance). Moreover, a constant Ornstein-Zernike-like contribution is needed to describe the data at large q_y_ values, accounting for fluctuations on small scale.

The fitted curves are shown as solid red lines in Fig. 2a. The half-sphere radii and distances are extracted from the fitting and shown in Fig. 2b,c, respectively. For films with thicknesses ≤35 nm, a broad peak I around q_y_ = 0.037 nm^−1^ (marked with the green solid arrow in Fig. 2a) is seen, which is the elongated (less ordered) PMMA nanostructure. The corresponding radius is about 52 nm and inter-domain distance is about 170 nm, respectively. For the film thickness ≥35 nm, another broad peak II is observed in the smaller q_y_ region (marked with the blue arrow in Fig. 2a), which indicates the structure of the PMMA half-spheres. As the film thickness increases from 35 nm to 56 nm, a layer of ordered half-spheres appears (AFM image, Fig. 1e), the inter-PMMA half-spheres’ distance decreases, resulting in the peak II moves to higher q_y_ value (shown in Fig. 2a). The corresponding radius remains constant at about 41 nm, while the inter-domain distance decreases from 225 nm to 172 nm. For the thickest film (91 nm), the peak II becomes more prominent, indicating that an improved order is formed. The improved order is related to the larger available space in vertical direction (larger film thickness) for the arrangement of the polymer chains inside the film. The corresponding half-sphere radius and distance remain constant at around 41 nm and 172 nm, respectively.

### The influence of SVA time

**Figure 3:**
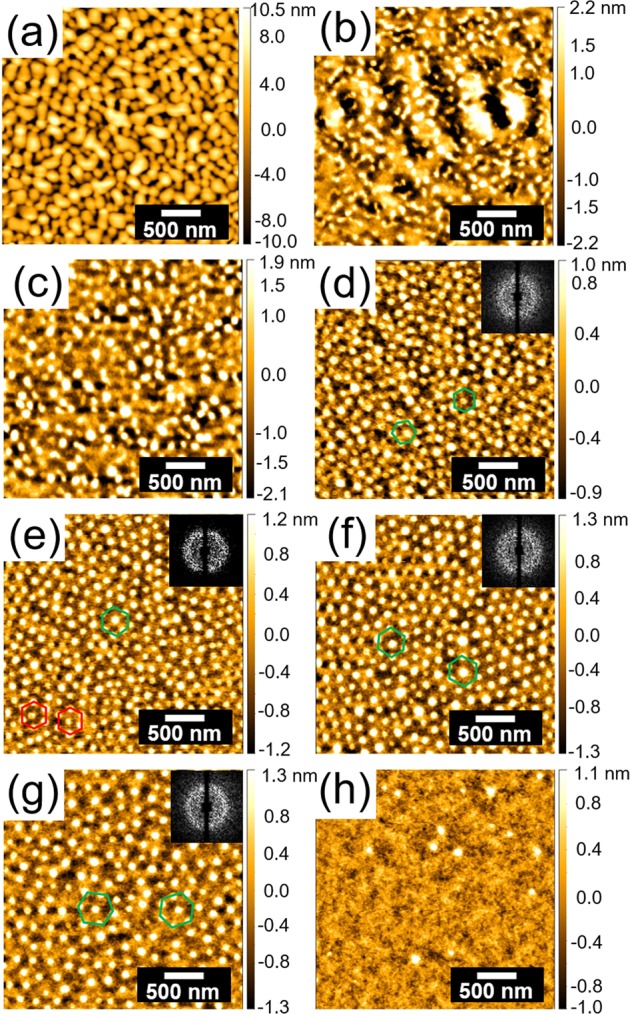
AFM images of PS-*b*-PMMA thin films with a thickness of 56 nm for different SVA times. (**a**) 0 h, (**b**) 0.5 h, (**c**) 1.0 h, (**d**) 2.0 h, (**e**) 3.5 h, (**f**) 5.5 h, (**g**) 18.0 h, and (**h**) 31.0 h. The height bar is adapted individually as indicated. The green hexagons indicate that the positions of these half-spheres are not well located at the apex of the hexagon. The red hexagons indicate that the positions of these half-spheres are well located at the apex of the hexagon. On the top right corners in (**d**–**g**), the insets show the corresponding 2D fast Fourier transform (FFT) patterns.

The influence of the SVA time on the morphology of the PS-*b*-PMMA films with a thickness of 56 nm is investigated at room temperature. Figure 3 shows AFM images of PS-*b*-PMMA thin films annealed in THF atmosphere for different times. The as-prepared film (Fig. 3a) displays a surface structure with large-sized continuous PMMA domains, since DMF is used for dissolving PS-*b*-PMMA, which is a good solvent for PMMA and a poor solvent for PS^9^. However, with increasing annealing time from 0 h to 0.5 h, the PMMA domain size decreases while the PS phase increases. This change in the surface morphology can be explained by the rearrangement of polymer chains due to the solvent vapor annealing using THF, which is a good solvent for both PS and PMMA. THF swells both blocks with slight PS selectively in the SVA process. As the annealing time increases from 0.5 h to 3.5 h, the morphology of the PS-*b*-PMMA films develops from a poorly ordered worm-like structure (Fig. 3b) to an half-sphere structure with local order (Fig. 3e). Moreover, a local hexagonal packing of half-spheres occurs when the annealing time is increased to 2.0 h (Fig. 3d, green hexagons). However, the hexagonal packing is irregular on large scale as seen from the ring pattern in the FFT analysis rather than having a 6-spot pattern. As the annealing time increases from 2.0 h to 3.5 h, the packing becomes locally more ordered, which can be confirmed by the red hexagons in Fig. 3e. This transition can be explained by the enhanced mobility of long polymer chains in the swollen BCP films, which allows the polymer chains to approach the thermodynamically preferred arrangement^10,27,51–54^. For the annealing time of 3.5 h, a locally better ordered spherical structure can be obtained. From the surface morphology, one can see that the average half-sphere diameter is about 78 ± 13 nm (Fig. S7e), and the average inter-domain distance is about 157 ± 20 nm (Fig. S7m). As the annealing time increases, further swelling and chain stretching continues, which leads to an increasing inter-domain distance to 189 ± 22 nm (Fig. S7o) for an annealing time of 18.0 h. The hexagonal packing is still irregular on large scale as indicated by the ring-like FFT pattern shown as inset in Fig. 3e–g. Moreover, the position of these half-spheres becomes more and more deviated from the apex of the hexagon in Fig. 3(e–g). However, the PS-*b*-PMMA film annealed for 31.0 h (Fig. 3h) shows a dramatic disappearance in the spherical morphology. This change can be ascribed to the dynamic change of interfacial interactions and commensurability conditions caused by the continual solvent absorption^3,10,48^. During the SVA process, THF vapor is preferentially absorbed by the PS surface layer due to slight selectivity of THF towards PS (χ_PS-THF_ < χ_PMMA-THF_)^20^. Therefore, part of the initially present PMMA half-spheres migrate into the film and PS dominates the film surface after a sufficient long annealing time, which is confirmed by the corresponding AFM phase image shown in Fig. S8b. The underlying process will be explained in more detail in the GISAXS analysis.

**Figure 4:**
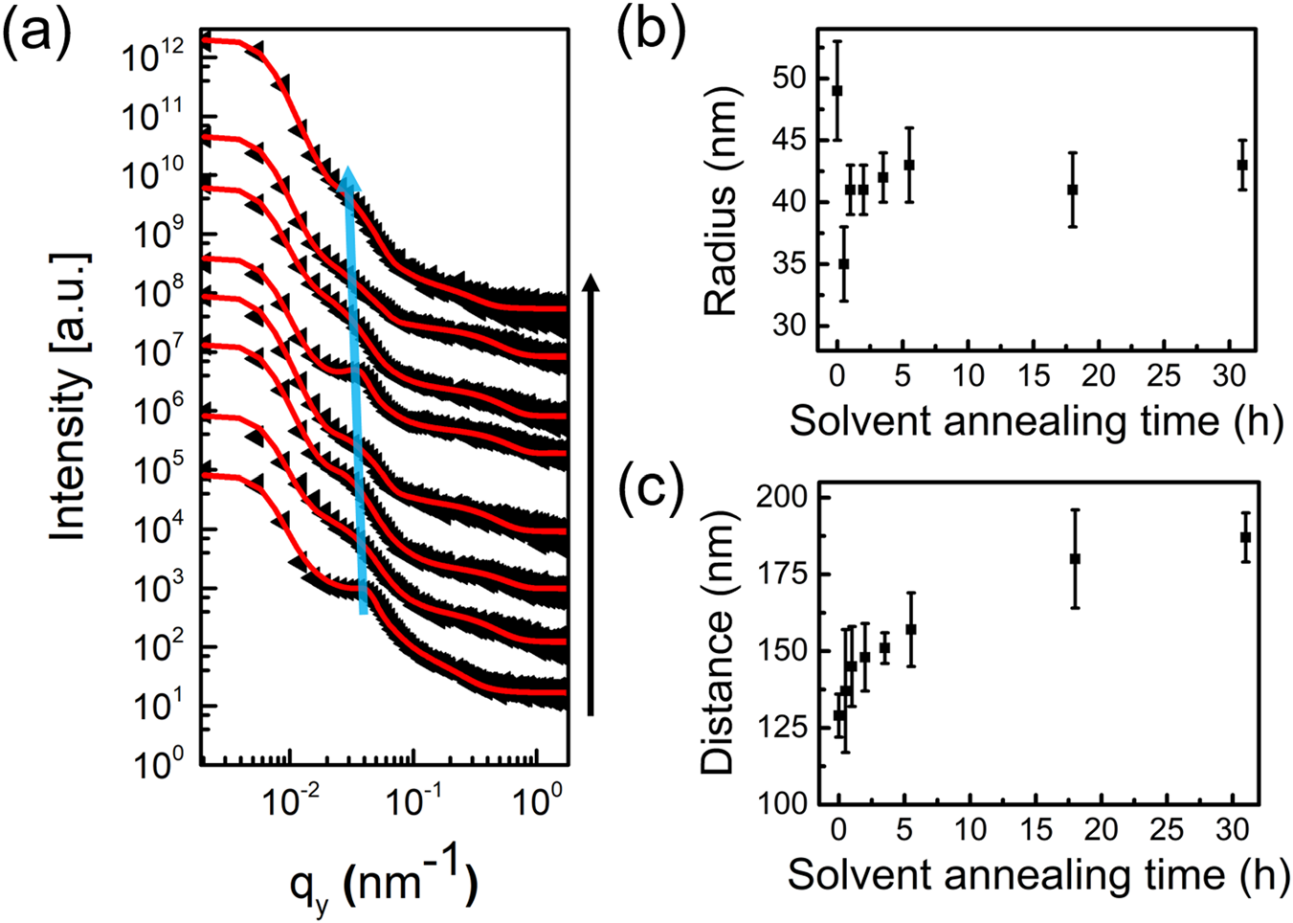
(**a**) Horizontal line cuts (q_y_) from the 2D GISAXS data of the PS-*b*-PMMA thin films (56 nm) with increasing SVA time. Data (black dots) and fits (solid red lines) are shifted vertically for clarity of the presentation (0 h, 0.5 h, 1.0 h, 2.0 h, 3.5 h, 5.5 h, 18.0 h and 31.0 h from bottom to top). The blue solid arrow serves as a guide to the eye for the peak. Half-sphere (**b**) radius and (**c**) distance of PS-*b*-PMMA nanostructures determined from the fits of GISAXS data plotted as a function of SVA time.

Figure 4 shows the horizontal line cuts (q_y_) from the corresponding 2D GISAXS data (Fig. S9) of PS-*b*-PMMA thin films after various SVA times. The curves are displayed together with their corresponding fits (solid red lines) in Fig. 4a. A broad peak (marked by the blue arrow) is observed. For the fitting of the horizontal line cut, the model as described before is used. The results of the extracted PMMA half-sphere radii and distances are depicted in Fig. 4b,c, respectively. 5c.

For the non-solvent annealed reference sample, a shoulder-like feature around q_y_ = 0.048 nm^−1^ is observed. This peak becomes weaker, and it moves towards lower q_y_ values with increasing the annealing time to 0.5 h, which indicates the formation of less ordered nanostructures with larger inter-domain distance at the onset of annealing. The corresponding average inter-domain distance increases slightly from around 129 nm to 137 nm (Fig. 4c) while the average half-sphere radius decreases from around 49 nm to 35 nm (Fig. 4b). Such a decrease of the half-sphere size is caused by the rearrangement of the polymer chains, which occurs when spin coated PS-*b*-PMMA films are placed in a new solvent-vapor atmosphere as we discussed above in AFM analysis (Fig. 3a,b). As the SVA time increases from 0.5 h to 3.5 h, the peak moves slightly to lower q_y_ values and becomes more prominent (Fig. 4a). This change indicates the formation of a higher ordered structure with larger inter-domain distance with increasing the annealing time. The corresponding average inter-domain distance and half-sphere radius increases from around 137 nm to 151 nm (Fig. 4c) and from around 35 nm to 41 nm (Fig. 4b), respectively, since the degree of micro-phase separation in swollen BCP films is improved by the enhanced chain swelling and the increased mobility under THF solvent vapor^10,53^. However, as the annealing time further increases from 3.5 h to 18.0 h, the peak becomes weaker while it still shifts to lower q_y_ values. This shift is the result of a continuous extension of the polymer chains, especially for the PS block segments. Since the DBC used has a PS volume fraction of 93%, the PS chain stretching has not reached maximum, which leads to the larger inter-domain distance distribution. The corresponding average inter-domain distance increases from around 151 nm to 180 nm (Fig. 4c) while the average half-sphere radius remains constant at around 41 nm (Fig. 4b). Prolonging the annealing time (from 18.0 h to 31.0 h) results in a PS chain stretching close to equilibrium and a more prominent peak is seen in the line cuts at an annealing time of 31.0 h (Fig. 4a). This indicates the formation of a higher ordered structure inside the film, which results from the rearrangement of polymer chains, causing part of the initially present PMMA half-spheres migrate into the film as discussed above in AFM analysis. The corresponding average inter-domain distance increases slightly from around 180 nm to 187 nm (Fig. 4c) while the average radius remains constant at around 41 nm (Fig. 4b) as before. The obtained radius of 41 nm indicates that the inner PMMA morphology is not full sphere, since the film thickness is only around 56 nm, which is not favorable to form full spheres with diameters of 82 nm. In our case, ellipsoid inside the film might be a reasonable morphology. Because at a swelling ratio of 1.7 (the initial 56 nm film swells to 91 nm where the nanostructure is in equilibrium), the swollen film thickness (around 91 nm) is sufficient to accommodate a full sphere (diameter of 82 nm from GISAXS analysis) that may get “squished” into an ellipsoid upon solvent evaporation. Moreover, earlier work from Tang *et al*.^38^ shows that the half-sphere were formed only on the sample surface.

### Evolution of morphology

**Figure 5:**
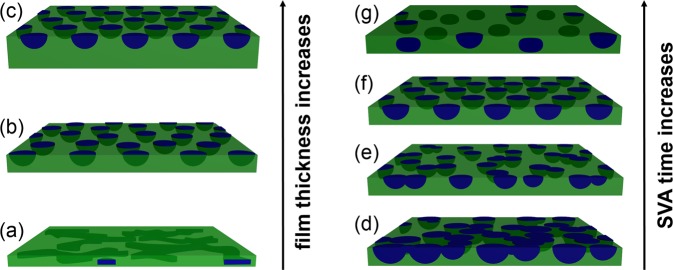
Schematic model of the microstructure formation of PS-*b*-PMMA thin films (left) as function of film thickness and (right) as function of SVA time. The PS phase (green) forms the matrix in which PMMA domains (blue) are embedded.

To illustrate the entire morphology evolution of PS-*b*-PMMA thin films, a schematic model is shown in Fig. 5 summarizing the findings of the AFM and GISAXS data analysis. On the left side of Fig. 5, the schematic model illustrates the evolution of the film morphology with different thicknesses after SVA in THF for 18.0 h. At small film thicknesses (Fig. 5a), a disordered structure inside the film is observed. This is the result of the confinement effects as observed with AFM measurements^55,56^. Moreover, the film thickness is too small to support the formation of full-spheres or even half-spheres. At a medium film thickness (higher than 0.5 D, lower than 0.5 L_0_, Fig. 5b), vertical half-spheres with the poorly hexagonal packing fully cover the substrate. This is a typical arrangement to allow the characteristic structure (L_0_) to be achieved in lateral direction. However, a similar structure with half-spheres aligned more ordered on the surface is seen at large film thicknesses (Fig. 5c), instead of full-spheres. Since in the case where the film thickness is close to 0.5 L_0_, a morphology with half-spheres is favorable for achieving the characteristic structure (L_0_) in lateral and vertical direction.

The right side of Fig. 5 shows the morphology evolution at various SVA times at room temperature in case of the film with a thickness of 56 nm. In case of the PS-*b*-PMMA thin film without any SVA (Fig. 5d), a structure with a continuous PMMA phase appears due to the PS-*b*-PMMA thin film was fabricated under non-equilibrium conditions with the spin coating method^57^. As THF diffuses from the atmosphere into the film, a decrease in size and increase of the number of PMMA half-spheres occurs (Fig. 5e). This is mainly attributed to the enhanced mobility of the polymer chains and a morphological rearrangement inside the thin films^10,33^. However, at this stage, there are still some island-like PMMA structures due to insufficient SVA time for highly entangled chain conformations in UHMW PS-*b*-PMMA thin films. When the film is treated in THF vapor for a certain time (i.e., 3.5 h), ordered half-spheres with hexagonal packing are obtained (Fig. 5f). Thus, an annealing time of 3.5 h is sufficient for the self-assembly of the UHMW polymer chains. However, when the PS-*b*-PMMA film is annealed further, PS dominates the free surface. Part of the initially present PMMA half-spheres migrate into the film (Fig. 5g) and become ellipsoids with same radius in film plane to minimize the energy of the system. This is confirmed by the AFM (surface morphology is PMMA half-spheres) and GISAXS analysis (inner morphology is PMMA ellipsoids).

## Conclusion

We report an approach for the fabrication of ordered, large-sized nanostructured thin films using spin coating of an asymmetric linear UHMW PS-*b*-PMMA DBC combined with SVA. Over a large concentration range, we observe a linear dependence of the film thickness on the polymer concentration in the solution used for spin coating, which before was known for polymers with smaller molecular weights. By applying an asymmetric UHMW DBC, we are able to tune the center-to-center distance of neighboring PMMA half-spheres to values larger than 150 nm with SVA (THF) time of 3.5 h. As a function of film thickness (≤0.5 L_0_), the morphology changes from disorder to relativity ordered PMMA half-spheres (locally regular hexagonal packed) in a PS matrix. This is the result of the confinement effects between the film thickness and inter-domain distance. With increasing SVA time, a rapid morphology evolution of locally hexagonal packed half-spheres (from poorly ordered to locally ordered, and then back to poorly ordered nanostructure) is observed, suggesting that a suitable SVA time is crucial for the formation of ordered nanostructures also in case of the UHMW DBC. Furthermore, when the annealing time is increased to 31.0 h, PS dominates the entire film surface.

The obtained DBC-based large-sized nanomaterials will pave the way for future applications. For example, using the UHMW DBC will provide sufficient space for embedding large-sized nanoparticles in addition to large inter-domain distance for application of photonic band gap structures (in the field of visible light).

## Methods

### Synthesis of DBC polystyrene-*block*-poly(methyl methacrylate)

An asymmetric DBC polystyrene-*block*-poly(methyl methacrylate), denoted as PS-*b*-PMMA, was synthesized *via* anionic block copolymerization of styrene and methylmethacrylate in tetrahydrofuran (THF). The number average molecular weight (*M*_n_) of PS-*b*-PMMA and the polydispersity (*Đ*), as characterized by size exclusion chromatography (SEC), were 1062 kg/mol and 1.15, respectively. The volume fraction of PMMA (*θ*_PMMA_) is 7%. Thus, a spherical nanostructure of the DBC is favorable, which is confirmed by our previous study on thick DBC films (1 mm) of this block copolymer^25^. Details of the PS-*b*-PMMA synthesis and the characterization protocols are described in the SI (Fig. S2, Table S1 and S2).

### Preparation of PS-*b*-PMMA thin films

The PS-*b*-PMMA films were prepared by spin coating at 1200 rpm for 60 s in the presence of flowing nitrogen (N_2_) gas. The nitrogen pressure was set to 1.8 bar for ensuring a better evaporation of the solvent DMF. In order to adjust the film thickness, DBC solutions in DMF with various concentrations (1.0, 3.0, 5.0, 10.0, 15.0 and 20.0 mg/mL) were applied to the acid cleaned silicon substrates (Si 100, Silchem, 15 × 15 mm^2^). Details for cleaning the silicon substrates were described in our previous article^58^. Here, in combination with the reported SVA time of 10 hours^32^ and ensuring sufficient time for self-assembly of UHMW DBC films, all the above-obtained films were annealed in solvent vapor (THF) for 18.0 h. To investigate the influence of SVA on the film structure, eight films with identical film thickness were prepared. Before SVA, all obtained films were dried at room temperature for 24 h to minimize the amount of residual solvent (DMF).

### Solvent vapor annealing (SVA)

Tetrahydrofuran (THF; high purity, Sigma-Aldrich) was used as a good solvent vapor for both blocks of PS-*b*-PMMA, which exhibits a small selectivity to PS. A desiccator (volume, V = 942.3 cm^3^ and surface area, S = 519.7 cm^2^) was used for THF vapor atmosphere creation. Inside the desiccator, a porous plate, on which the samples were placed, was positioned over the liquid solvent. The desiccator was tightly sealed with PARAFILM® M (Carl Roth GmbH + Co. KG), which consists mostly of polyolefin and paraffin waxes. The whole SVA process was carried out at room temperature and the films were swelling approximately by a factor of 1.7. After SVA, all films were removed from the desiccator as quickly as possible to allow for a preservation of the obtained nanostructures^59^, rather than deswelling the films at slow rates^60^.

### Characterization techniques

The film thickness values were measured via surface profilomery (DektakXT Stylus Profiler, Bruker) and X-ray reflectivity (XRR, D8-advance, Bruker). To examine the surface morphology of PS-*b*-PMMA thin films, atomic force microscopy (AFM, MFP-3D, Asylum Research) equipped with a sharpened tip (curvature radius of 7 nm) was operated in tapping mode to probe the nanoscale morphology. To study the inner film structure, grazing incidence small-angle X-ray scattering (GISAXS) measurements were performed at the Austrian SAXS beamline of the Elettra synchrotron source with a wavelength of 1.54 Å (energy, E = 8 keV) in Trieste, Italy. For obtaining a desirable q-range, the grazing incidence angle and the sample-detector distance were set to 0.40° and 2.083 m, respectively. A 2D detector (Pilatus3 1M with pixel size of 172 × 172 µm^2^, 981 by 1043 pixel array) was used to detect the scattering signal.

## Supplementary information

supplementary information

## Data Availability

The datasets generated during and/or analyzed during the current study are available from the corresponding author on reasonable request.
