# Supplementary material for: Self-Assembly in ultrahigh molecular weight sphere-forming diblock copolymer thin films under strong confinement
Source: Sci Rep. 2019 Dec 4;9:18269. doi: 10.1038/s41598-019-54648-3 (PMC6892843; doi:10.1038/s41598-019-54648-3)
Supplement: Supplementary file 1 — supplementary information [file 41598_2019_54648_MOESM1_ESM.pdf]

Supporting Information

Self-Assembly in ultrahigh molecular weight sphere-forming diblock copolymer thin films under strong confinement

*Wei Cao[1], Senlin Xia[1], Michael Appold[2], Nitin Saxena[1], Lorenz Bießmann[1], Sebastian Grott[1], Nian Li[1], Markus Gallei[3], Sigrid Bernstorff[4], Peter Müller-Buschbaum[1,5] **

[1]Technische Universität München, Physik-Department, Lehrstuhl für Funktionelle Materialien, James-Franck-Straße 1, 85748 Garching, Germany

[2]Technische Universität Darmstadt, Ernst-Berl-Institute for Technical and Macromolecular Chemistry, Alarich-Weiss-Straße 4, 64287 Darmstadt, Germany

[3]Saarland University, Chair in Polymer Chemistry, Campus C4 2, 66123 Saarbrücken, Germany

[4]Elettra-Sincrotrone Trieste S.C.p.A., Strada Statale 14, km 163.5, in AREA Science Park, 34149 Trieste, Italy

[5]Technische Universität München, Heinz Maier-Leibnitz Zentrum (MLZ), Lichtenbergstr. 1, 85748 Garching, Germany

Email: muellerb@ph.tum.de

Experimental Section for synthesis of a polystyrene-block-poly(methylmethacrylate)

Reagents. All solvents and reagents were purchased from Alfa Aesar, Sigma Aldrich, Fisher Scientific, ABCR and used as received unless otherwise stated. Deuterated solvents were purchased from Deutero GmbH, Kastellaun, Germany. Tetrahydrofuran (THF) was distilled from sodium/benzophenone under reduced pressure (cryo-transfer) prior to the addition of 1,1-diphenylethylene and *n*-butyllithium (*n*-BuLi) followed by a second cryo-transfer. Styrene and methylmethacrylate (MMA) were dried by stirring over calcium hydride (CaH_2) or trioctylaluminium and cryo-transferred prior to use. All syntheses were carried out under an atmosphere of nitrogen using Schlenk techniques or a glovebox equipped with a Coldwell apparatus.

Instrumentation. NMR spectra were recorded on a Bruker DRX 300 spectrometer working at 300 MHz (^1H NMR). NMR chemical shifts are referenced relative to tetramethylsilane. Standard SEC was performed with a system composed of a 1260 IsoPump - G1310B (Agilent Technologies), a 1260 VW - detector - G1314F - at 254 nm (Agilent Technologies) and a 1260 RI-detector - G1362A - at 30 °C (Agilent Technologies), THF as the mobile phase (flow rate 1 mL min^{-1}) on a SDV column set from PSS (SDV 10^3, SDV 10^5, SDV 10^6). Calibration was carried out using PS standards (from Polymer Standard Service, Mainz). For data acquisition and evaluation of the measurements, PSS WinGPC® UniChrom 8.2 was used. For determining the thermal properties of the polymers differential scanning calorimeter (DSC) was performed with a Mettler Toledo DSC-1 in a temperature range from 0 °C to 150 °C with a heating rate of 10 K min^{-1}.

Anionic Block Copolymerization of Styrene and Methylmethacrylate

Exemplary synthesis of a polystyrene-block-poly(methylmethacrylate) featuring a molar mass of 1223 kg/mol (PS_{10193}-b-$PMMA_{951}$)[1]

In an ampule equipped with a stirring bar, 10 mg (0.24 mmol, 260 eq.) LiCl and 760 mg (7.26 mmol, 8013 eq.) neat styrene were dissolved in 60 mL of dry THF. The solution was cooled to -78 °C before the polymerization was initiated by quick addition of 70 µL (0.91 µmol, 0.013 M solution in hexane, 1 eq.) *s*-BuLi with a syringe. After 1 h an aliquot of the solution is taken from the ampule for characterization of the PS segment and determined by adding methanol. Then, 64 µL (1.81 µmol, 0.028 M solution in hexane, 2 eq.) DPE is added to the active macroanions and the solution is stirred for 1 h at room temperature. After the solution was cooled for 1 h at -80 °C again 150 mg (1.50 mmol, 1653 eq.) MMA were added to the living chains and the solution was stirred for 24 h at -80 °C. After adding a small amount of degassed methanol, the polymer was poured into a 10-fold excess of water. The polymer was collected by filtration, washed with water and dried in vacuum (yield: 815 mg, 90 %).

SEC (vs PS): PS: M_n = 994 700 g mol^{-1}; M_w = 1 061 600 g mol^{-1}; $Đ$ = 1.07

PS-*b*-PMMA: M_n = 1 061 600 g mol^{-1}; M_w = 1 222 700 g mol^{-1}; $Đ$ = 1.15

^{1}H-NMR (300 MHz, 300 K, $CDCl_3$, δ in ppm): 7.20-6.90 (br, $H_{4/5}$), 6.79-6.34 (br, H_3), 3.61 (br, H_8), 2.21-0.84 (alkyl).

XRR data and film thickness

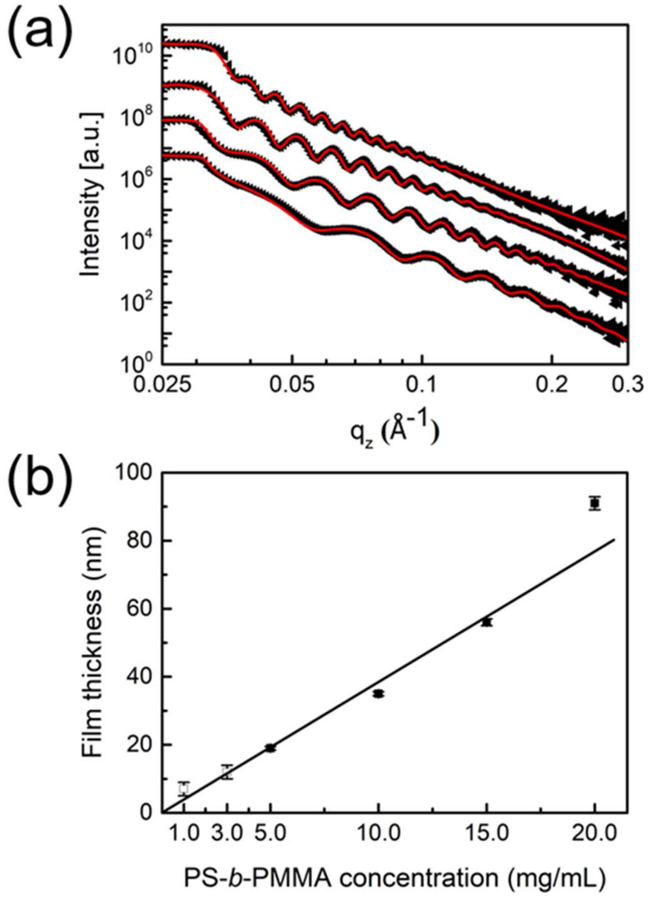

Figure S1. a) XRR data (symbols) with fits (red solid lines) of PS-*b*-PMMA films produced with different concentrations. Curves from bottom to top refer to PS-*b*-PMMA films with concentrations of 5.0, 10.0, 15.0 and 20.0 mg/mL DBC in DMF. b) Film thickness plotted as a function of PS-*b*-PMMA concentration as measured by profilometry (□) and XRR (■). The solid line represents a linear correlation of film thickness and PS-*b*-PMMA concentration.

Polymer characterization

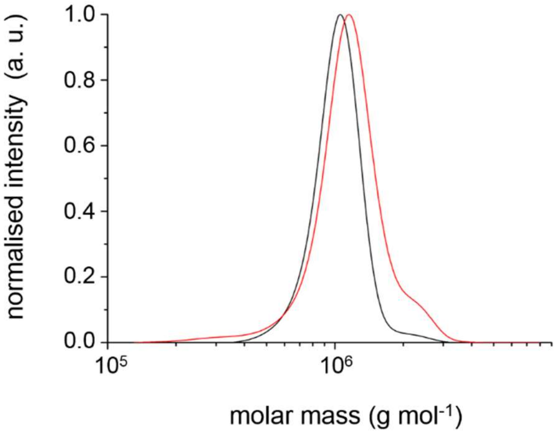

Figure S2. Molar mass distributions obtained by SEC measurements vs. PS standards in THF obtained for the PS_{10193}-precursor (black line) and PS_{10193}-*b*-$PMMA_{951}$ (red line). The signal area integration yield 1.76 wt% of PS dimer and 5.46 wt% of diblock copolymer dimer, calculated by using the elution volume and via Gauß approximation. The coupling of polymer is caused by some reactions with the carbonyl functionality in PMMA. For the dimers also appear in PS homopolymer, the reason might be that traces of oxygen were present in the termination reagent (methanol).

Table S1. Characterization data of block copolymer in this study.

	Polymer	M_n^a	M_w^a	M_n^b	$Đ^a$
1	PS_{10193}	994.7	1061.6	-	1.07
2	PS_{10193}-*b*-$PMMA_{951}$	1061.6	1222.7	1156.8	1.15

[a)] molecular weight determined by SEC in kg mol^{-1} (PS standards, THF), [b)] molecular weight determined by ^{1}H NMR spectroscopy in kg mol^{-1}.

Table S2. The parameters of BCP used in this study with respect to molar ratio x, weight ratio w and volume ratio Φ.

	Polymer	$x_{PS/PMMA}$	$w_{PS/PMMA}$	$\Phi_{PS/PMMA}$
1	PS_{10193}-*b*-$PMMA_{951}$	91/9	92/8	93/7

[a] The molar, weight and volume fractions were calculated by combining SEC (PS) and NMR (PS-*b*-PMMA) and using the densities of 1.04 g cm^{-3} and 1.18 g cm^{-3} for PS and PMMA, respectively.

Distribution of PMMA half-sphere size/distance for large film thicknesses

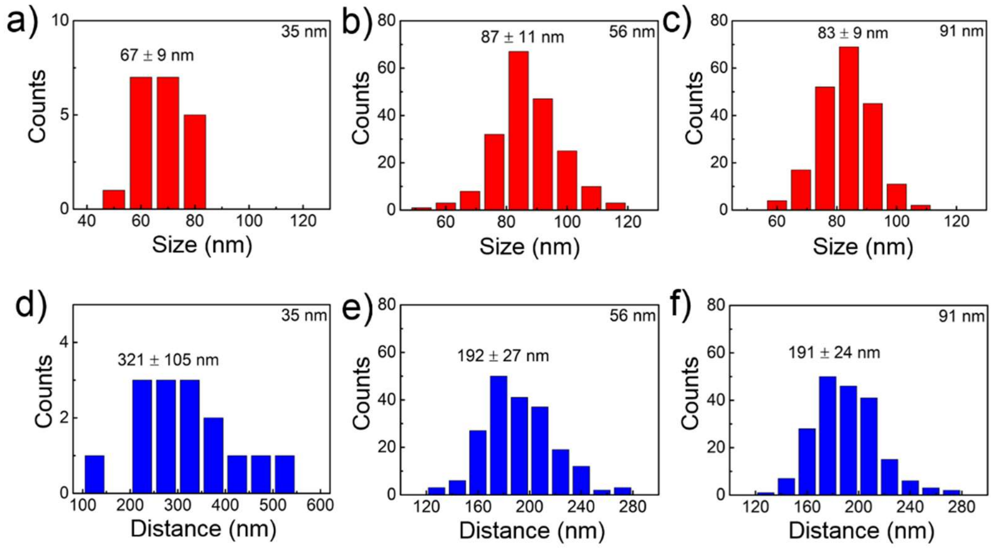

Figure S3. Half-sphere size distribution (red bar chart) and distance distribution (blue bar chart) of PS-*b*-PMMA thin films (SVA time is 18.0 h) with thickness of a + d) 35 nm, b + e) 56 nm, c + f) 91 nm. Distance is the distance between neighboring PMMA half-spheres. All of the data are calculated statistically from the corresponding AFM images by using Image J 1.46r.

AFM phase images of PS-*b*-PMMA thin films with different thicknesses

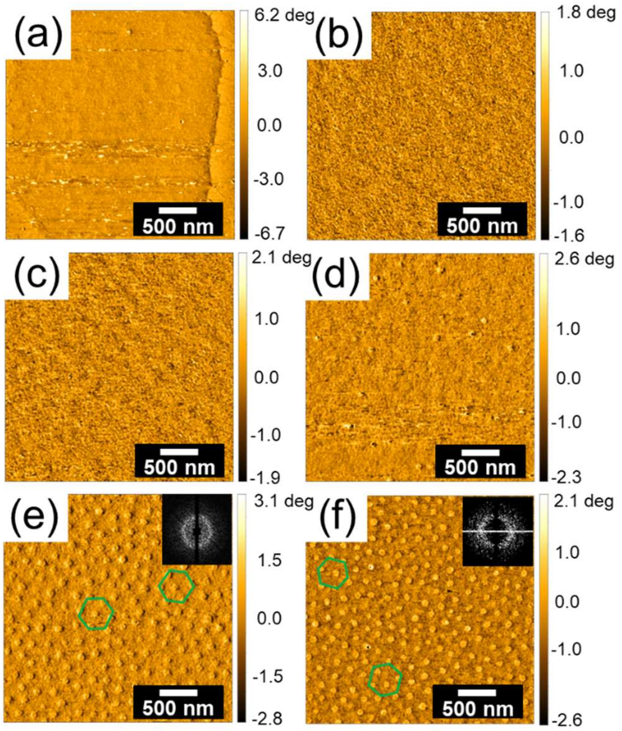

Figure S4. AFM phase images of PS-*b*-PMMA thin films with thickness of (a) 7 nm, (b) 12 nm, (c) 19 nm, (d) 35 nm, (e) 56 nm, and (f) 91 nm after SVA in THF for 18 h. The green hexagons indicate that the positions of these half-spheres are not well located at the apex of the hexagon. On the top right corners, the insets show the corresponding 2D fast Fourier transform (FFT) patterns.

GISAXS data and modelling

The effective interface approximation (EIA) of the distorted wave Born approximation (DWBA) was used, which is a simplistic, widely and successfully used approach. As common in small angle scattering, we describe the diffuse scattering factor $P_{diff}(\vec{q})$ in terms of form factor $F(\vec{q})$ and structure factor $S(\vec{q})$ to model scattering objects (form factor) in a certain distance (structure factor), assuming N objects

$$P_{diff}(\vec{q}) \propto NS(\vec{q})F(\vec{q})$$

We select the so-called local monodisperse approximation (LMA). The LMA is based on the assumption that in local domains, which have the size of the coherence length of the beam, only monodisperse objects, i.e. of one size, are found. In our modeling, a spherical geometry of the scattering objects is assumed to match the AFM observations. There is no difference in the film plane between full sphere model and half sphere model. From the fitting we obtain average object sizes and distances. For a quantitative analysis of the lateral structure inside the films, horizontal line cuts of the 2D GISAXS data were performed along the q_y direction (shown as the red rectangle in Figure S5) at the Yoneda peak position via the software DPDAK.[2] Afterwards, the extracted horizontal line cuts were fitted as the above description.

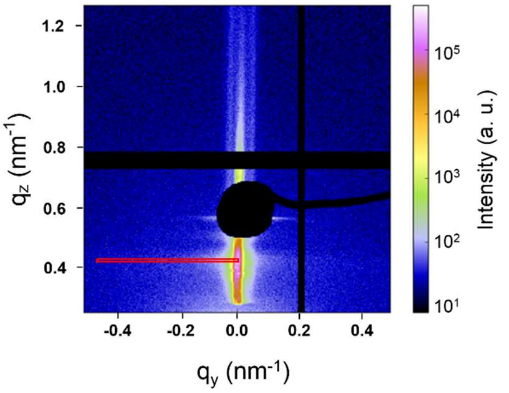

Figure S5. Horizontal line cut was done along the q_y direction (shown as the red rectangle) at the Yoneda peak position. The specular peak is shielded by a beamstop.

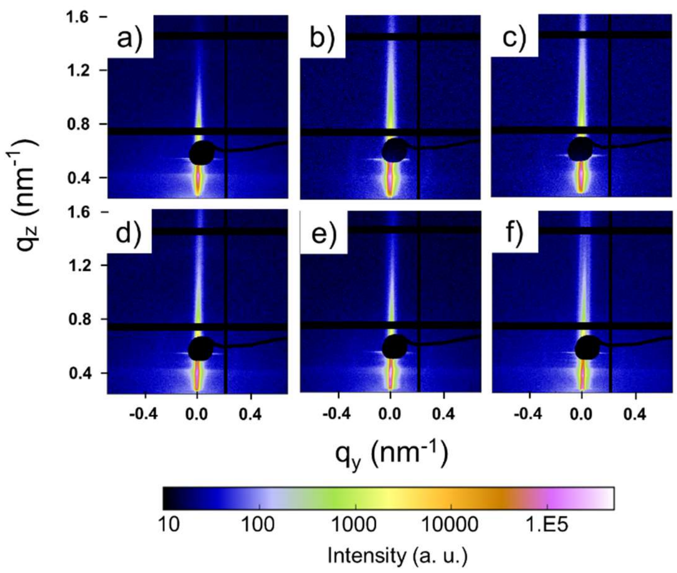

Figure S6. 2D GISAXS data of PS-*b*-PMMA thin films with thickness of a) 7 nm, b) 12 nm, c) 19 nm, d) 35 nm, e) 56 nm, and f) 91 nm after SVA in THF for 18 h. The specular peak is shielded by a beamstop.

Distribution of PMMA half-sphere size/distance for different solvent annealing times

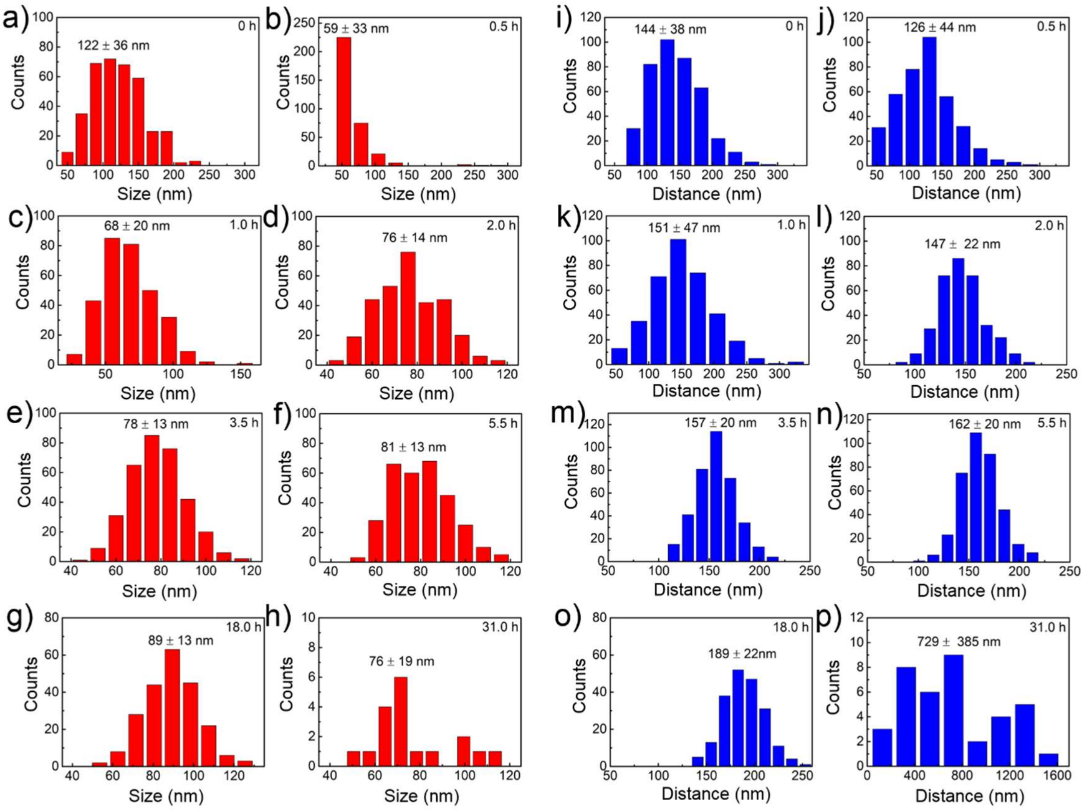

Figure S7. Half-sphere size distribution (red bar chart) and distance distribution (blue bar chart) as a function of solvent annealing time in PS-*b*-PMMA thin films (56 nm). a) and i) 0 h, b) and j) 0.5 h, c) and k) 1.0 h, d) and l) 2.0 h, e) and m) 3.5 h, f) and n) 5.5 h, g) and o) 18.0 h, h) and p) 31.0 h. For disordered phase consecutive structures (a, b and c), Half-sphere size is the domain length for two orthogonal directions. For micro-phase separated half-spheres (d, e, f, g and h), half-sphere size is the domain diameter. Half-sphere distance is the distance between neighboring domains. All of the data are calculated statistically from the corresponding AFM images by using Image J 1.46r, with a total of at least 200 half-spheres or inter-domain distances being calculated on each image. For the PS-*b*-PMMA thin films with annealing time of 31.0 h, the amount of count is far less than 200 due to many half-spheres migrate inside the film.

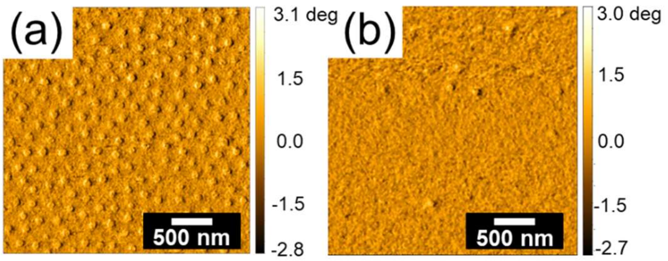

Figure S8. AFM phase images of PS-*b*-PMMA thin films with a thickness of 56 nm for different SVA times: a) 18.0 h and b) 31.0 h.

2D GISAXS images of PS-*b*-PMMA films with various SVA times

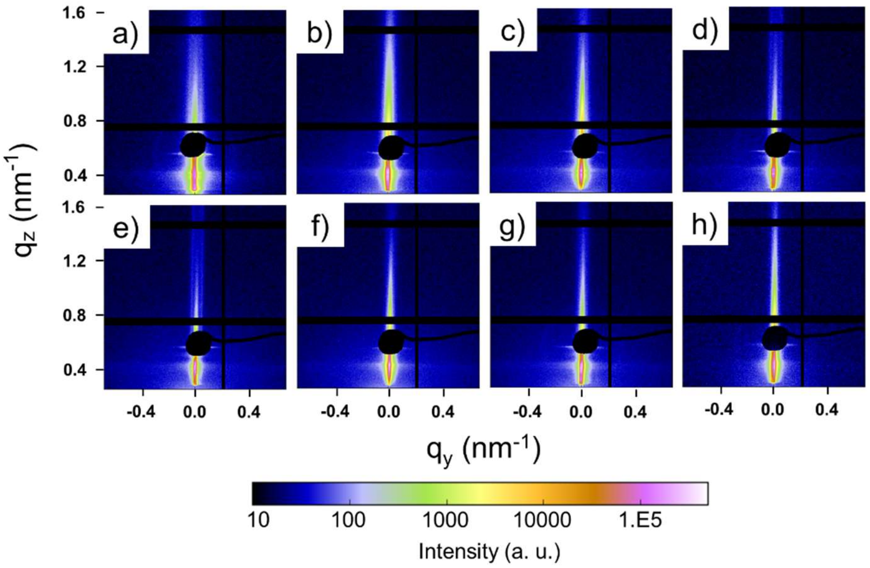

Figure S9. 2D GISAXS data of PS-*b*-PMMA thin films with a thickness of 56 nm for different SVA times. a) 0 h, b) 0.5 h, c) 1.0 h, d) 2.0 h, e) 3.5 h, f) 5.5 h, g) 18.0 h, and h) 31.0 h. The specular peak is shielded by a beamstop.

REFERENCE

1. Appold, M.; Gallei, M. Bio-Inspired Structural Colors Based on Linear Ultrahigh Molecular Weight Block Copolymers. *ACS Appl. Polym. Mater.* **2019**, *1*, 239-250.
2. Benecke, G.; Wagermaier, W.; Li, C.; Schwartzkopf, M.; Flucke, G.; Hoerth, R.; Zizak, I.; Burghammer, M.; Metwalli, E.; Müller-Buschbaum, P.; Trebbin, M.; Förster, S.; Paris, O.; Roth, S. V.; Fratzl, P. A Customizable Software for Fast Reduction and Analysis of Large X-Ray Scattering Data Sets: Applications of the New DPDAK Package to Small-Angle X-Ray Scattering and Grazing-Incidence Small-Angle X-Ray Scattering. *J. Appl. Crystallogr.* **2014**, *47*, 1797-1803.
